# Managing genetic diversity in breeding programs of small populations: the case of French local chicken breeds

**DOI:** 10.1186/s12711-022-00746-2

**Published:** 2022-08-03

**Authors:** Gwendal Restoux, Xavier Rognon, Agathe Vieaud, Daniel Guemene, Florence Petitjean, Romuald Rouger, Sophie Brard-Fudulea, Sophie Lubac-Paye, Geoffrey Chiron, Michèle Tixier-Boichard

**Affiliations:** 1 Université Paris Saclay, INRAE, AgroParisTech, GABI, 78350 Jouy-en-Josas, France; 2 Centre INRAE Val de Loire, UMR-BOA, SYSAAF, 37380 Nouzilly, France; 3 Centre INRAE Val de Loire, UMR-BOA (UR83), 37380 Nouzilly, France; 4 Centre de Sélection de Béchanne, Hameau de Béchanne, 01370 Saint-Etienne-Du-Bois, France; 5 ITAVI, 23 rue Jean Baldassini, 69364 Lyon Cedex 07, France

## Abstract

**Background:**

On-going climate change will drastically modify agriculture in the future, with a need for more sustainable systems, in particular regarding animal production. In this context, genetic diversity is a key factor for adaptation to new conditions: local breeds likely harbor unique adaptive features and represent a key component of diversity to reach resilience. However, local breeds often suffer from small population sizes, which puts these valuable resources at risk of extinction. In chickens, population management programs were initiated a few decades ago in France, relying on a particular niche market that aims at promoting and protecting local breeds. We conducted a unique comprehensive study of 22 French local breeds, along with four commercial lines, to evaluate their genetic conservation status and the efficiency of the population management programs.

**Results:**

Using a 57K single nucleotide polymorphism (SNP) chip, we demonstrated that both the between- and within-breed genetic diversity levels are high in the French local chicken populations. Diversity is mainly structured according to the breeds’ selection and history. Nevertheless, we observed a prominent sub-structuring of breeds according to farmers’ practices in terms of exchange, leading to more or less isolated flocks. By analysing demographic parameters and molecular information, we showed that consistent management programs are efficient in conserving genetic diversity, since breeds that integrated such programs earlier had older inbreeding.

**Conclusions:**

Management programs of French local chicken breeds have maintained their genetic diversity at a good level. We recommend that future programs sample as many individuals as possible, with emphasis on both males and females from the start, and focus on a quick and strong increase of population size while conserving as many families as possible. We also stress the usefulness of molecular tools to monitor small populations for which pedigrees are not always available. Finally, the breed appears to be an appropriate operational unit for the conservation of genetic diversity, even for local breeds, for which varieties, if present, could also be taken into account.

**Supplementary Information:**

The online version contains supplementary material available at 10.1186/s12711-022-00746-2.

## Background

During the last decades, climate change has been demonstrated to occur as a result of human activities and to have a growing impact [1]. Climate change will have multiple consequences on the availability of water and food resources but also on the distribution of diseases and of their vectors, putting many parts of the world at risk [2]. This will impact agricultural production, which will have to adapt to these new climatic conditions to ensure food security, in particular in more threatened regions of the globe that often already suffer from limited food supplies [3]. Thus, emphasis should be put on diversified and low input production systems that will take advantage of locally available resources, such that they become less dependent on imported products [4]. Indeed, climate change mainly results from greenhouse gas emissions, such as carbon dioxide or methane, and can be mitigated by reducing these emissions [5]. Animal breeding contributes significantly to these emissions, which means that domestic animals both suffer from and enhance climate change. In addition, many ethical concerns have been raised about animal breeding, which means that more effort should be made to improve animal welfare to fulfil consumers expectations, for example through free-range farming [6]. Thus, it is necessary to develop a more sustainable model for genetic improvement of animals that will be able to adapt to new farming (e.g. free-range, local feed, etc.) and climatic conditions (e.g. high temperature). In this context, genetic diversity is considered to be essential by conserving the adaptive potential of livestock [7].

Poultry are among the most important contributors to animal production worldwide, with the highest increase in output of all livestock species over the last decades, in particular in developing countries [8]. Following domestication, chicken populations have evolved towards a large diversity of breeds, some of them being submitted to intense selection in order to be profitable in large-scale production systems [9]. In particular, selection of poultry has led to the specialization of distinct breeds for either egg (layers) or meat (broilers) production, since growth and reproduction traits are antagonistic [10]. Compared to ancestral populations, commercial lines, which are the most strongly selected, show a deficit in rare alleles [11]. Yet, compared to layer lines, within-breed diversity has remained relatively high in broiler lines [12], which could be due to founder effects and different selection practices. At the same time, many traditional local breeds have become at risk of disappearing, although they may still harbor original genetic variants at key loci.

In this context, considering the impact that global change could have on breeding goals with respect to climate, resources or diseases, traditional local poultry breeds may represent a valuable reservoir of genetic diversity for future breeding [2]. Yet, their population sizes are often limited, making them prone to inbreeding and strong genetic drift. As a result of competition from more productive breeds, they have very likely experienced drastic and recent bottlenecks, due to a decrease in their use. Consequently, many local breeds are at the edge of extinction [13] and molecular surveys have revealed that some traditional breeds have retained high diversity whereas others have not [12, 14, 15]. Consequently, careful management of these populations is critical to cope with future challenges to livestock production.

In France, local breeds often have an important heritage value and are often named after the name of the region or city they originated from. Some of these local breeds have benefited from the development of designations of origins or quality brands since the 1950s (e.g., Protected Origin for Bresse chicken) and the 1960s (e.g. ‘Label rouge’, a quality specification) as documented by [16]. These quality certifications guarantee high-quality products with controlled processes of breeding, including breed identification, free-range production, locally identified production of slow-growing animals, and an older age at slaughter than in intensive production systems. In this context, since a few decades, initiatives have been set up to develop management programs for local poultry breeds, including pedigree recording, optimized mating plans and moderate selection.

In this study, we conducted a large and unique survey of 24 representative French local chicken populations using both molecular (57K single nucleotide polymorphism (SNP) genotyping) and demographic (pedigree and management features) data to (i) evaluate the genetic diversity status of French breeds and (ii) assess the impact of population management programs that have been put in place on genetic diversity. For comparison purposes, we also included data from four commercial lines to provide a comprehensive view of the diversity patterns associated with different kinds of population management. Based on such an assessment, we identify some lessons and make recommendations for the management of genetic diversity that could be extended to any genetic conservation program. In particular, we highlight the suitability of molecular tools to monitor the genetic diversity of local populations for which pedigree information is scarce.

## Methods

### Breed management and sampling strategy

The aim of the choice of the local breeds included in our study was to represent the diversity of origins and localities of French breeds as much as possible. These populations are generally described by a standard phenotype that is a marker of their identity. These breeds usually do not compete with each other since they are often associated with a specific niche market in a particular region. Breeding goals are defined collectively at the breed association level based on weight, laying rate, etc. Nevertheless, the standard of a breed is an important part of the breeding goals and is maintained based on phenotypic traits such as body shape, body size, feather color, and comb shape. Only individuals that have extreme phenotypes or morphological defects are eliminated.

We distinguished three groups of breeds based on population management (see Table 1 that also lists all local breeds with their code name, geographic origin, morphological standard, and historical records). Group 1 includes 18 French local breeds and 20 populations that are part of a management program with pedigree recording. For 18 of these populations, a nucleus flock was sampled in the Breeding Center of Béchanne (CSB; (http://centrebechanne.fr/) in 2013. The CSB is in charge of the breeding program for 15 populations (ALS, B11, B22, B55, BAR, BOU, CHA, CNF, GAS, GAT, GG, GN, GOU, HOU, and NDB) and of the conservation program for four breeds (CDR, GDT, GDV and MER). The CSB has the full responsibility of the breeding program for eight of the 15 breeds, (B11, B22, B55, GN, GG, HOU, CHA, and CNF) and acts on behalf of the breed associations for the others. Thus, the nucleus flocks at the CSB are representative of each breed or population and are used to provide chicks to farmers for small-scale production. Breeding units consist of one sire mated with three to four unrelated hens (based on available pedigrees). At collection time, eggs are individually identified according to the mother hen, to be able to construct the pedigrees. A particular effort was made to try to maintain almost all the initial sire and hen families that were present at the creation of the flock across generations. Group 1 also included a selected population of the Marans breed (MAG), which consists of three lines bred by a single breeder for commercial production of eggs with a typical dark brown eggshell. All breeding schemes and mating plans were designed with SYSAAF, a professional union (https://www.sysaaf.fr/sysaaf_eng/), that is also in charge of data storage and management (phenotypes and pedigrees). SYSAAF is in charge of the estimation of breeding values, with the aim to limit inbreeding rate and coancestry of the populations for its members (i.e. CSB, other breeds’ associations, and the MAG breeder). Tailor-made solutions have been developed to optimize breeding schemes and mating plans in order to limit inbreeding rates while applying selection. They rely on a constrained optimization procedure based on simulated annealing [17]. Thanks to this organization, we could obtain precise pedigree data for 18 of the 20 breeds from the SYSAAF database, with the written agreement of each breed’s association. The numbers of sires and dams at sampling time and at the start of each program are in Table 2, together with the year when pedigree recording started, ranging from 1989 to 2009 depending on the breed. Although breeding goals were declared by CSB and breed associations for most of these breeds, among which for the culling of very slow-growing animals, selection intensity was not precisely documented but appeared to be moderate. No information was provided for the MAG population but we expected a stronger selection pressure for this breed than for the others in Group 1 due to their larger commercial market.

**Table 1:** Sampling design and population information

Breed name	Breed code	Group	Number of animals	Geographic area in France	Center of origin mean coordinates	Historical records	Plumage color
Poule d’Alsace	ALS	1	30	North East	48.57 N; 7.75 E	Nineteenth century	Black
Barbezieux	BAR	1	59	South-West	45.47 N; − 0.15 W	Nineteenth century	Black
Bourbonnaise	BOU	1	57	Centre	46.57 N; 3.34 E	n.a	Silver
Bresse Gauloise Blanche B11	B11	1	60	Centre-East	46.21 N; 5.23 E	Ancestral Bresse (sixteenth century)	White
Bresse Gauloise Blanche B22	B22	1	60	Centre-East	46.21 N; 5.23 E	Created in 2000 from B11	White
Bresse Gauloise Blanche B55	B55	1	43	Centre-East	46.21 N; 5.23 E	1950 (egg production)	White
Charollaise	CHA	1	56	Centre-East	46.44 N; 4.28 E	1950 (meat)	White
Contres	CON	2	41	Centre	47.42 N; 1.43 E	Twentieth century	Silver
Cou nu du Forez	CNF	1	59	Centre-East	45.74 N; 4.23 E	n.a	White
Coucou de Rennes	CDR	1	55	West	48.11 N; − 1.68 W	Nineteenth century	Sex-linked barring
Gasconne	GAS	1	60	South-West	43.43 N; 0.58 E	Nineteenth century	Black
Gâtinaise	GAT	1	57	South of Paris	48.15 N; 2.70 E	Fifth century	White
Gauloise Grise	GG	1	60	Centre-East	46.21 N; 5.23 E	Sixteenth century	Autosomal barring
Gauloise noire	GN	1	58	Centre-East	46.63 N; 5.22 E	Sixteenth century, currently bred for egg production	Black
Geline de Touraine	GDT	1	59	Centre-West	47.13 N; 1.00 E	Fifteenth century	Black
Gournay	GOU	1	58	West	49.48 N; 1.72 E	Nineteenth century	Mottled
Grise du Vercors	GDV	1	44	South-East	45.02 N; 5.29 E	Created in 2002 from a cross	Sex-linked barring
Houdan	HOU	1	58	West of Paris	48.79 N; 1.60 E	Seventeenth century	Mottled
Hergnies	HER	2	60	North	50.48 N; 3.52 E	Recent reconstitution	Autosomal barring
Le Mans	MAN	2	29	Centre-West	48.01 N; 0.20 E	Recent reconstitution	Black
Marans managed	MAG	1	52	Centre-West	47.62 N; − 1.19 W	Commercial breeding for eggs	Brown red, red, silver cuckoo,
Marans fancy	MAR	2	65	Centre-West	46.3 N; − 1.0 W	Fancy breeders, first described in fifteenth century	Black, Brown red, red, silver cuckoo, white, wheaten, wild-type,
Merlerault	MER	1	38	West	48.7 N; 0.29 E	Nineteenth century	Black
Noire du Berry	NDB	1	56	Centre	46.95 N; 1.99 E	Nineteenth century	Black

Number of animals genotyped (Nb_anim), mean geographic location, history of creation, and main morphological features for French local breeds that were engaged in a management program (Group 1) or distributed among fancy breeders (Group 2)

**Table 2:** Pedigree data available for managed local populations (Group 1)

Breed	Number of male founders	Number of female founders	First year with pedigree	Number of generations in 2013	Number of sires in 2013	Number of dams in 2013	Mean inbreeding coefficient in 2013 (%)	Pedigree-based Ne in 2013 (Ne_demo)	Initial founder-based Ne (Ne_found)	Inbreeding rate per generation in % (Delta_F)
Poule d’Alsace (ALS)	20	33	2008	5	27	74	2.3	65	49.8	0.46
Barbezieux (BAR)	7	7	2002	11	52	116	7.0	27	14	0.66
Bourbonnaise (BOU)	75	232	2005	8	40	100	2.4	114	226.7	0.30
Bresse Gauloise Blanche B22	26	102	2000	13	99	215	5.3	108	82.9	0.42
Bresse Gauloise Blanche B11	23	256	1989	24	105	346	7.1	122	84.4	0.31
Bresse Gauloise Blanche B55	23	255	1989	24	104	376	nd	153	84.4	NA
Charollaise (CHA)	34	103	2005	8	20	43	2.7	63	102.3	0.34
Cou nu du Forez (CNF)	34	98	2007	6	20	50	3.5	53	101	0.59
Gasconne (GAS)	5	5	2009	4	24	77	7.2	22	10	1.85
Gâtinaise (GAT)	11	12	2006	7	40	112	5.6	56	23	0.82
Gauloise Grise (GG)	77	252	1998	15	65	184	NA	285	235.9	NA
Gauloise Noire (GN)	108	275	1996	17	20	83	5.3	119	310.2	0.32
Géline de Touraine (GDT)	43	103	1996	17	55	110	4.2	142	121.3	0.25
Gournay (GOU)	24	61	2003	10	46	104	nd	98	68.9	0.46
Grise du Vercors (GDV)	29	83	2007	16	24	53	2.7	65	86	0.27
Houdan (HOU)	30	81	2004	9	24	58	NA	80	87.6	NA
Merlerault (MER)	18	32	2006	7	14	34	5.6	44	46.1	0.82
Noire du Berry (NDB)	27	47	2008	5	47	103	2.7	99	68.6	0.55

The pedigree population size (Ne_demo) was calculated according to [19] and the mean inbreeding rate was obtained by dividing the mean inbreeding coefficient by the number of generations since the start of the management program. Marans MAG population data were not available since it was managed by a commercial breeder. NA stands for non-available data

Group 2 involved four local breeds (CON, HER, MAN, and MAR) that have not yet been included in a management program with pedigree recording, so these animals were sampled from two to six independent farms, depending on the breed, from 2013 to 2014, and no pedigree information was available. One of these populations corresponds to the Marans chickens (MAR) that are raised by fancy breeders and have the same origin and similar eggshell color as the MAG population of Group 1. However, a larger number of color varieties was observed in the Marans flocks kept by six fancy breeders, who are motivated by the conservation of this breed with no large commercial production.

Group 3 included four commercial lines as control populations to assess breed identity and possible introgression events. For these purposes, DNA samples from two fast-growing broiler lines (FG1 with 42 individuals and FG2 with 43 individuals) were obtained from the DNA collection that was set up for the Aviandiv EU project in 1999 [18] and were genotyped in this study. Genotyping data were also obtained for one line of a French slow-growing high quality ‘label’ chicken, SGB, (96 individuals) and for one line of a brown-egg layer, LAY, (57 individuals). A data transfer agreement was established with the owners of these lines in order to re-use existing genotyping data without re-sampling, but no phenotypic data and pedigree data were provided.

The average number of individuals studied per population was 56, and ranged from 29 for the MAN breed to 96 for the ‘label’ chicken line SGB, with a total of 1512 individuals across breeds. Family structure was considered in order to sample distinct families as much as possible. Blood and DNA samples for 1350 animals of the 24 local populations (Group 1 + Group 2) were stored under the project name BioDivA at the @BRIDGe biological resource center of the CRB-Anim infrastructure (CRB-Anim, https://doi.org/10.15454/1.5613785622827378E12), and a material transfer agreement was signed with each breed association and each individual farmer.

### Data analyses

Using management program information, we estimated effective population sizes either (i) at the start of the program from the number of founders and their sex-ratio (${Ne}_{founders}$) using the classical formula, ${Ne}_{founders}=\frac{4NmNf}{Nm+Nf}$, where $Nm$ and $Nf$ are the number of sires and dams, respectively, or, (ii) at the date of sampling (2013) for breeds maintained by CSB in Group 1, using complete pedigrees (${Ne}_{demo}$) according to Cervantes et al*.* [19]. Individual inbreeding coefficients, ${F}_{pedig}$, were also estimated using the available pedigree data. Considering that coancestry and inbreeding were set to null at the start of the pedigree, we computed the mean inbreeding rate, $\Delta F$, following Falconer and MacKay [20], $\Delta F=1-{(1-{\overline{F} }_{pedig})}^{1/n}$, with $n$ the number of generations in the pedigree and ${\overline{F} }_{pedig}$ the mean inbreeding coefficient.

Genotypes were obtained using the 57K Illumina Beadchip [21]. After mapping on the current version of the chicken genome assembly (Gal_gal_6), we kept 51,041 SNPs with a single identified location in the genome. Then, we applied quality control filters using the Plink 1.9 software [22], requiring a minor allele frequency (MAF) higher than 0.001 (*–maf* function) and call rates of at least 5% for both markers and individuals (*–mind* and *–geno* functions). This resulted in a set of 46,940 autosomal SNPs for 1512 individuals, with a total call rate of 99.86%.

For some analyses, we also constructed a pruned dataset from which linked markers were removed using the Plink 1.9 software by applying the *–indep-pairwise* function for sliding windows of 30 SNPs and a step size of 10 SNPs, and a r^2^ threshold of 0.8 above which markers were considered in high linkage-disequilibrium. This resulted in a pruned dataset of 45,209 autosomal SNPs.

The level of diversity at the population level was investigated by computing six summary statistics with the Plink 1.9 software: (i) the mean individual inbreeding coefficient computed using allele frequencies estimated across all populations and all groups, (ii) the mean individual inbreeding coefficient computed using allele frequencies estimated for each population, (iii) the mean observed heterozygosity, (iv) the expected heterozygosity, (v) the average MAF, and (vi) the proportion of fixed alleles in the population. The two first indices were computed using the method of moments estimation procedure [23] on the pruned dataset (*–het* function) as follows $F=\frac{{\#Hom}_{obs}-{\#Hom}_{exp}}{\#loci-{\#Hom}_{exp}}$, with ${\#Hom}_{obs}$, ${\#Hom}_{exp}$ and $\#loci$, the number of observed and expected homozygous loci and the total number of non-missing loci, respectively. These two first indices, which are inbreeding coefficients estimated using allele frequencies computed either at the whole or population levels, are very similar to Fit and Fis [24], respectively, under the assumption that deviations from Hardy–Weinberg equilibrium are only due to inbreeding, and thus will referred to as Fit and Fis, hereafter. Global Fst values based on the *–fst* function of the Plink 1.9 software were also computed on the pruned dataset between populations and within populations according to breeders or lines for MAN, HER, MAG and MAR.

Runs of homozygosity (ROH) were computed using the *–homozyg* function of the Plink 1.9 software with the following parameters: a minimum ROH size of 500 kb, a minimum number of 30 SNPs in a ROH, a density of at least one SNP every 50 kb, and allowing for one missing and heterozygous SNP per window of 50 SNPs. The proportion of ROH in the genome was converted into inbreeding coefficients following MacQuillan et al. [25].

For the Group 1 breeds, pairwise correlations were computed for genetic diversity measures of the populations with each management program feature (${Ne}_{founders}$, initial number of sires, initial number of hens, and number of generations since the management program has been in place). Since the four management program features are correlated to each other, we used partial correlations using the ggm package [26] to correct for the three other features when testing one program feature, except for ${Ne}_{founders}$, which was only corrected for number of generations (${Ne}_{founders}$ being calculated based on the numbers of hens and sires). Pairwise correlations were also computed between all population diversity estimates and management program features.

Genetic distances between all individuals were computed as an identity-by-state distance matrix (*–distance square ibs* function of plink 1.9), which was then used to compute and plot an unrooted neighbor-joining tree using the APE R package [27]. The tree plots for the MAR and HER populations were made using the ggtree R package [28] in order to include additional information such as the breeders and the phenotype (namely feathers’ colors).

Pairwise genetic distances between populations were computed as Nei’s distance D or Fst using the Stampp R package [29]. Nei’s pairwise D distances were then used to compute a neighbor-net tree of populations using the Splitstree software [30]. Correlations of pairwise Fst with geographical distances were tested with a Mantel test using the Vegan R package [31].

In a previous study, 14 French local breeds were separated into two groups according to their genetic similarity with Asian breeds, as a result of the importation of Asian breeds in the nineteenth century [14]. In order to search for this structure in the dataset of the current study, we investigated population structure using a discriminant analysis on principal components (DAPC), using the ADEgenet R package [32, 33], with the number of clusters K set to 2. The probability for a given breed to belong to each cluster was plotted based on the breed’s geographic origin (French departments) in order to reveal possible centers of dissemination following the introduction of Asian breeds, and plotted on a map with sampling locations.

All additional computations and graphical representations were done using R (R Core Team, 2019) and the emmeans [34], ggplot2 [35], and corrplot [36] R packages.

## Results

### Demographic parameters

**Fig. 1:**
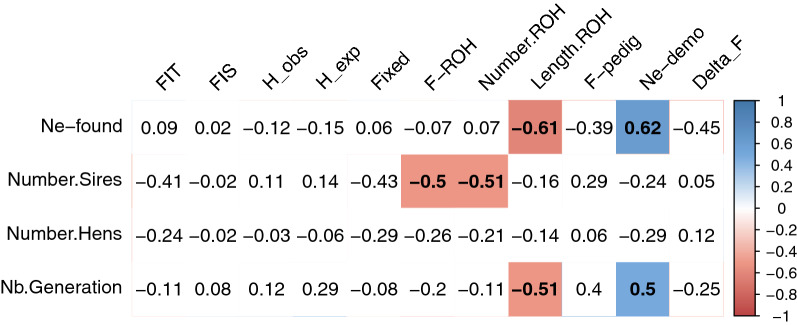
Partial correlation matrix between current population diversity indices and management program features for breeds of Group 1. Values correspond to partial Pearson’s correlation coefficients. Each correlation was corrected by management program features that are not involved in the correlation, except for correlations involving ${Ne}_{founders}$, which were only corrected for the number of generations. Colored cells stand for significant correlations (p < 0.05), either positive (blue) or negative (red); color intensity reflects the strength of the correlation; white cells represent non-significant correlations. “Nb.Generation” stands for the number of generations since the start of the management program and “Fixed” is the proportion of fixed alleles

Estimates of ${Ne}_{demo}$ ranged from 22 to 285 for the 18 breeds from Group 1 (Table 2). Interestingly, the populations with the smallest ${Ne}_{demo}$ estimates had implemented a population management program relatively recently. The mean year of onset of the management program was 1996 for breeds with a ${Ne}_{demo}$ larger than 100 and 2006 for breeds with a ${Ne}_{demo}$ smaller than 100. Considering that the generation interval is generally one year, the populations with the largest number of generations since the onset of the management program tended to exhibit a larger ${Ne}_{demo}$, with a significant positive correlation (r = 0.5) between ${Ne}_{demo}$ and the number of generations since the onset of the program, when correcting for the number of sires and hens at the start of the program (Fig. 1). The corrected correlation of the effective population size at the start of the program, ${Ne}_{founders}$, with the inbreeding rate, $\Delta F$, was negative and almost significant (r = − 0.45; p = 0.07). The mean ${Ne}_{founders}$ at the start of the program was 100 but varied greatly between populations, ranging from 10 for the GAS breed to 310 for the GN breed (Table 2). ${Ne}_{demo}$ was greater than ${Ne}_{founders}$ for 11 populations, particularly for the GG and Bresse B55 breeds, but was lower for seven populations, particularly for the GN and BOU breeds. The correlation between ${Ne}_{demo}$ and ${Ne}_{founders}$ was significant and positive, at 0.62, when correcting for the number of generations (Fig. 1). The mean pedigree-based inbreeding coefficient at sampling was small, at 4.5% and ranged only from 2.3 to 7.2%. For most populations, the ratio of female to male founders ranged from 1 to 4, but was as high as 11 for two populations (Bresse Gauloise blanche B11 and B55).

### Molecular information

#### Genetic diversity

**Fig. 2:**
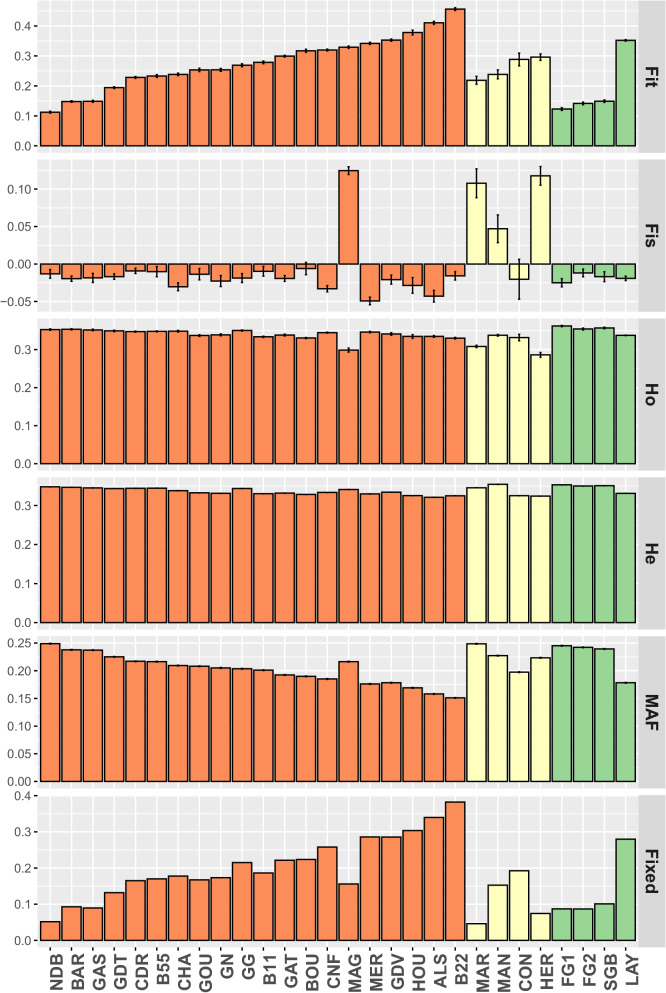
Genetic diversity summary statistics per population. From the top to the bottom, Fit, Fis, observed heterozygosity (Ho), expected heterozygosity (He), minor allele frequency (MAF), and proportion of fixed alleles (Fixed). Populations were sorted by group (Group 1 in orange, Group 2 in yellow, and Group 3 in green) and by Fit within groups. Error bars correspond to standard errors

The within-population genetic diversity was characterized by the mean of six indices (Fig. 2) and (see Additional file 1: Table S1). The mean Fit ranged from 0.11 for the NDB breed to 0.46 for the B22 breed, with a mean value of 0.26 (standard error 0.09), and the mean Fis ranged from − 0.049 for the MER breed to 0.12 for the MAG breed, with a mean value of − 0.003 (s.e. 0.009). The mean observed heterozygosity rate was 0.34 (s.e. 0.01) and ranged from 0.31 for the MAG breed to 0.36 for the B55 breed. The mean expected heterozygosity was homogeneous among the breeds, ranging from 0.32 for the ALS to 0.35 for the MAN breed, with a mean of 0.34 (s.e. 0.002). The mean MAF across breeds was 0.21 (s.e. 0.03) and ranged from 0.15 for the B22 breed to 0.25 for the NDB breed. Finally, the mean proportion of fixed alleles was 0.18 (s.e. 0.09) and ranged from 0.05 for the MAR breed to 0.38 for the B22 breed. Management groups differed only in terms of Fis and Ho, with breeds from Group 2 (local non-managed breeds) having higher and lower values, respectively, than those of the two other groups (see Additional file 2: Table S2) and (Fig. 2). Correlations between molecular and demographic estimates of genetic diversity for Group 1 are presented in Additional file 3: Fig. S1.

#### Runs of homozygosity

**Fig. 3:**
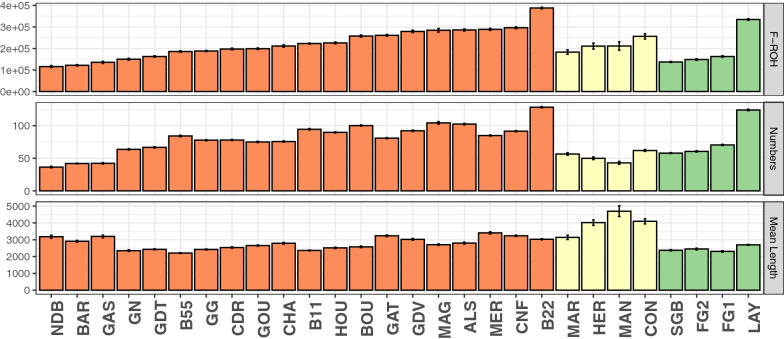
Runs of homozygosity (ROH) summary statistics per population. From top to bottom, inbreeding coefficient (F-ROH), mean number of ROH per individual, and mean length of each ROH. Populations were sorted by group (Group 1 in orange, Group 2 in yellow, and Group 3 in green) and by F-ROH within groups. Error bars correspond to standard errors

Runs of homozygosity (ROH) were detected and converted into inbreeding coefficients, F-ROH (Fig. 3) and (see Additional file 1: Table S1). The mean F-ROH ranged from 0.13 for the NDB breed to 0.42 for the B22 breed, with a mean across all breeds of 0.24 (s.e. 0.01). The mean length of homozygous segments was 2.9 Mb (s.e. 112 kb) and ranged from 2.2 Mb for the B55 breed to 4.7 Mb for the MAN breed. The mean number of ROH per individual was 76.3 (s.e. 4.5) and ranged from 36.6 for the NDB breed to 128.4 for the B22 breed. The three groups of breeds differed significantly only for the length of ROH, with populations from Group 2 having longer ROH (Fig. 3) and (see Additional file 2: Table S2).

#### Genetic structure

**Fig. 4:**
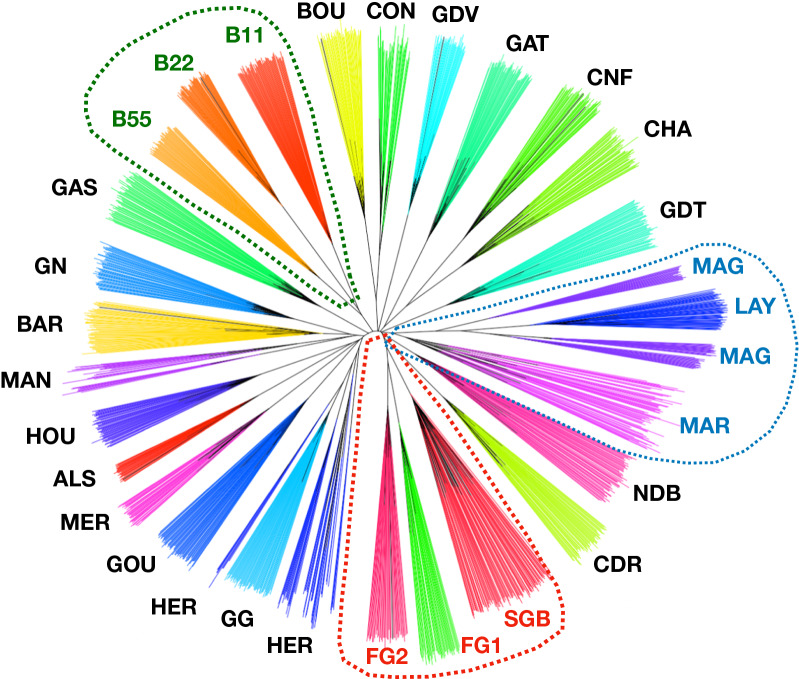
Unrooted neighbor joining tree of individuals with respect to populations. Dotted lines represent grouping features: brown egg laying breeds (blue), broiler breeds (red), and Bresse breeds (green)

*All populations* Most populations were genetically nearly completely separated and clearly distinguishable from each other (Fig. 4). The MAG population had three distinct groups, with the LAY population inserted in-between. Similarly, the HER population was separated into several groups, among which the GG population was inserted.

The mean weighted Fst between all populations was 0.26 (s.e. 5.7 $\times$ 10^–4^). Estimates of pairwise Fst were all significantly different from 0 (see Additional file 4: Fig. S2), and ranged from 0.12 between the MAR and NDB populations to 0.44 between the LAY and B22 populations.

**Fig. 5:**
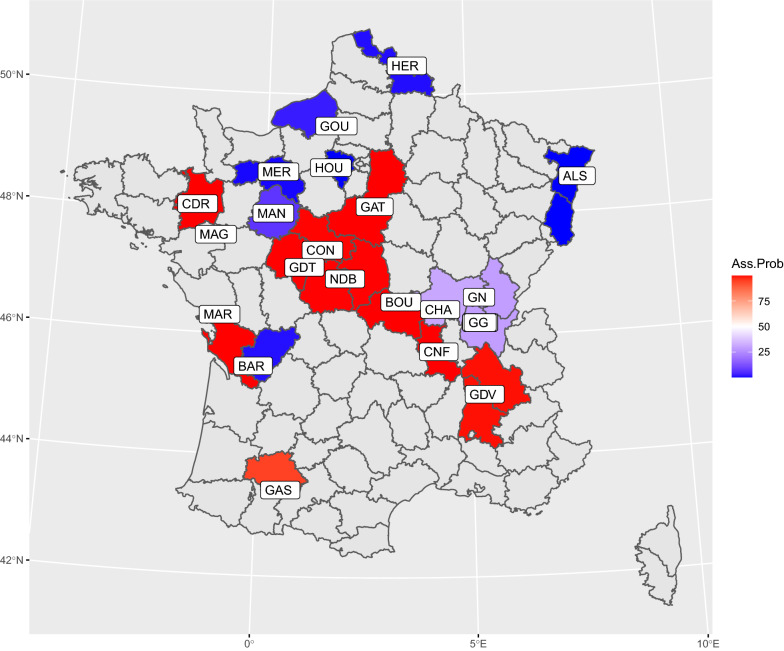
Mean center of origin of populations and cluster assignment of the regions. The color of each French region (“department”) corresponds to the mean assignment of their chicken populations to each of the two clusters of the DAPC, red for Asian and blue for European

**Fig. 6:**
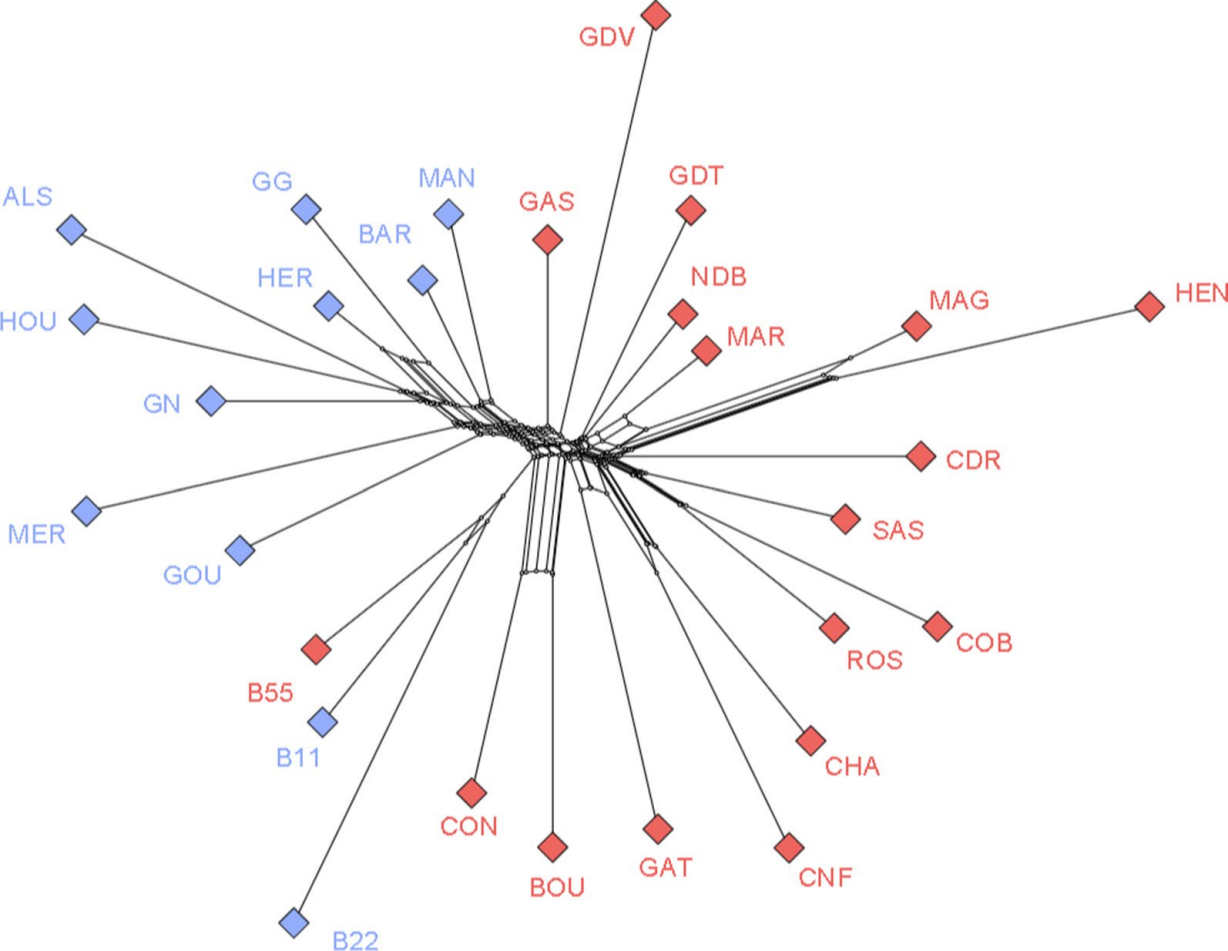
Neighbornet tree of the chicken populations. The color of each diamond corresponds to the population cluster affiliation, red for Asian and blue for European

The mean assignment probability to each of the two clusters in the DAPC was averaged for each population (see Additional file 5: Fig. S3) and showed that each population was clearly affiliated with a given cluster, except for the B55 population. Figure 5 shows the geographic location of breeds, together with the most probable cluster they belong to. The neighbornet tree (Fig. 6) confirmed the separation of the two clusters of origin, European and Asian, except in the case of the three Bresse populations (B11, B22, and B55), which were grouped together on the edge of the Asian cluster, although the B11 and B22 lines showed a higher probability of assignment to the European cluster. We also observed a close relationship between the MAG and LAY populations and between the HER and GG populations. The broiler populations were grouped together, fast-growing populations (FG1 and FG2) being closer to each other than to the slow-growing ‘label’ population (SGB). The Mantel test of the correlation between 1/(1-Fst) and the log of geographical distance was not significant.

*Within-breed genetic structure* Three breeds exhibited a within-population structure, the Marans (MAR and MAG), HER and MAN breeds. These breeds exhibited significant Fst values between the different flocks, representing either varieties or breeders, and are described in detail in the following.

**Fig. 7:**
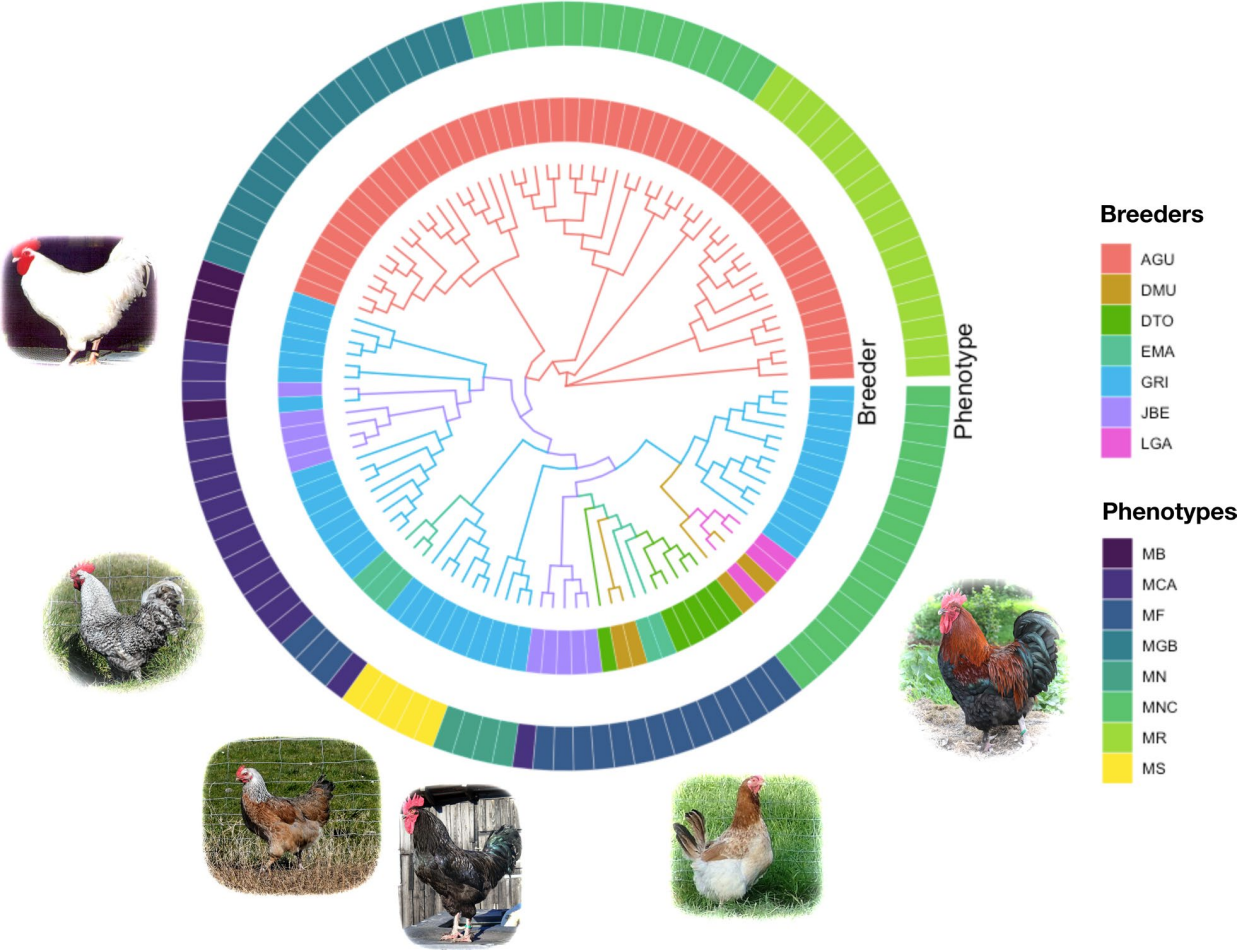
Unrooted neighbor-joining tree of the Marans breed. Colors of the surrounding circles corresponds to the breeders (inner circle) and the phenotypes (outer circle)

The MAR (Group 2) and the MAG (Group 1) subpopulations of the Marans breed were clearly separated, while the LAY population (Group 3) was surprisingly intermingled with the MAG group, for which different sublines could be distinguished. The Fst between the MAG and MAR clusters was 0.14 (s.e. 6.0 $\times$ 10^–4^). The global Fst between the seven breeders was 0.14 (s.e. 5.6 $\times$ 10^–4^), while that between the six breeders of the MAR group was 0.06 (s.e. 4.1 $\times$ 10^–4^). Another clustering was also observed for the Marans breed based on the phenotype of feather color, for which we observed an Fst of 0.17 (s.e. 5.7 $\times$ 10^–4^). When the MAR and MAG populations were considered separately, the between-color Fst was 0.11 (s.e. 5.1 $\times$ 10^–4^; 6 colors) for the MAR population and 0.18 (s.e. 7.4 $\times$ 10^–4^; 3 colors) for the MAG population, as shown in Fig. 7. The HER breed was also strongly structured, reflecting the six fancy breeders, with a corresponding Fst of 0.20 [s.e. 7.5 $\times$ 10^–4^; (see Additional file 6: Fig. S4)]. The MAN breed also exhibited a genetic structure that reflected the two fancy breeders, with a Fst of 0.18 (s.e. 9.5 $\times$ 10^–4^) between them.

## Discussion

### Structure and genetic diversity of local French chicken breeds

Population clustering generally depends on various factors, such as selection, geographical location, and life history traits. In the current study, the clustering of populations was influenced first by the relatively recent Asian introduction that was previously described [14]. This is consistent with the history of chickens in Europe, which are characterized by an initial dispersion from Asia in the Iron Age, followed by a second introduction of Asian breeds 150 to 200 years ago [37]. The presence of these two groups, one originating from the first introduction, named European, and one from the second Asian introduction named Asian, could be of interest in terms of selection perspectives if heterosis is observed in crosses between these groups, which would make them heterotic groups, which are widely used in plant breeding [38]. Thus, cross-breeding experiments are needed to investigate whether crosses between these local chicken breeds show heterosis.

A second level of clustering was observed according to breeding goals, with the three intensely-selected broiler populations (FG1, FG2 and SGB), the three free-range slow-growing Bresse lines under moderate selection ((B11, B22 and B55), and the subpopulations of the Marans breed clustering together with the commercial layers (MAR, MAG and LAY). Within the Bresse sub-cluster, the B22 line was the most inbred, which is due to its relatively smaller number of founder hens compared to the other Bresse lines (about 200 for B22 vs 350 for B55 and B11). In addition, the founders of B22 were chosen based on a specific phenotype, i.e. a pale comb color, which likely originates from a subset of families in the ancestral Bresse population. Finally, selection for a pale comb phenotype was applied in the B22 population only, which may have led to stronger genetic drift and inbreeding.

The most probable explanation for the clustering of LAY with the Marans breed, and more specifically the MAG lines, is that a crossbreeding event between commercial Rhode Island red layers and ancestors of the MAG lines occurred in the past, motivated by the need to improve the laying rate of these lines. However, crossbreeding between commercial layers and some Marans individuals cannot be excluded either, with the motivation to darken eggshell color of commercial Rhode Island red layers. A similar situation was observed with the GG and the HER breeds, because one fancy breeder of HER chickens introduced GG individuals in its breeding scheme to improve their phenotype and the genetic diversity of his flock.

Geographical clustering, as in the case of the CNF and CHA breeds, which both originate from the same French region, suggests a history of exchanges between neighbouring farmers at market places. However, other breeds from the same region do not exhibit such clustering, as could have been expected for the NDB and GDT breeds. Indeed, we did not find any sign of isolation by distance, probably due to multiple introductions and origins [37]. Historical records frequently report that the creation of new breeds often involved crossing between multiple populations, either local or introduced, specifically for a given phenotype which is often the basis for chicken breed characteristics (e.g. feather color, comb size…). Taken together, the French chicken breeds exhibit a high level of genetic diversity, with populations clearly differentiated from each other. The high level of genetic differentiation between breeds, with an overall Fst of 0.26, was comparable to what has been observed in other European countries, with an overall Fst of 0.25 for British breeds [39] and 0.22 for Hungarian breeds [40]. However, the observed genetic differentiation between French breeds is larger than that observed among Chinese populations, which had an overall Fst of 0.11 [41] and pairwise Fst ranging from 0.03 to 0.27 [42], compared to our estimates ranging from 0.12 to 0.44 for French local breeds. In contrast, French populations exhibited lower Fis values than those reported for ornamental breeds in Great Britain [39], Germany [43], and The Netherlands [15]. Expected heterozygosity values were very close to those observed for local Asian populations, ranging from 0.29 to 0.34 [42]. Interestingly, these levels of diversity usually correspond to less structured populations in which gene flow occurs regularly between breeds, ensuring the conservation of a high level of within-breed genetic diversity. In contrast, other European populations exhibit a strong genetic structure with limited gene flow between breeds but limited within-breed diversity due to very small population sizes, as they are often maintained by fancy breeders without a proper conservation program. Thus, it appears that high levels of genetic diversity have been preserved both within and between breeds for the local French chicken populations.

### Impact of management programs on genetic diversity

Our data allowed us to compare the impact of different breeding and management programs on genetic diversity. Diversity indicators did not differ significantly between the highly selected (Group 3) and the managed local breeds (Group 1), both groups exhibiting a high level of genetic diversity. In particular, Fis values were almost null for both groups 1 and 3, indicating good management of matings, regardless of the level of diversity maintained in these populations. This was surprising since we expected low levels of genetic diversity in the local populations due to their limited population sizes, resulting in genetic drift and inbreeding. One exception in Group 1 is the MAG population that consists of three separate lines, which suggest combined effects of isolation of each line, a limited number of founders, and a higher selection intensity. There was, however, a trend towards less fixed and rare alleles for the commercial than the local populations, although this difference was not significant probably because of the LAY population, as layer populations are often much less genetically diverse than broilers because of a smaller base population.

We did observe a significant difference in Fis between the unmanaged (i.e. Group 2) and the managed breeds (Groups 1 and 3), with higher positive Fis values for three managed breeds (MAR, MAN and HER), which can be explained by a Wahlund effect [44]. This subdivision of populations is due to the very few exchanges that occur between the different breeders for the HER and MAN breeds, as confirmed by the high subsequent Fst between the different flocks. In the case of the MAR breed, this within-breed genetic structure is mainly due to different varieties of feather color as also revealed by large Fst, while a moderate Fst was found between the flocks of the different breeders. One could argue that, for the same variety or breed, it is advisable to maintain multiple subpopulations, instead of one large population, in order to conserve more genetic diversity and more alleles. Indeed, this question of conserving a single large or several small populations (i.e. the SLOSS debate) is largely debated in ecology at the species level [45]. Here we found no difference in terms of expected heterozygosity with the other groups, which supports the hypothesis that diversity can be conserved similarly in single large or in small sub-structured breeds. However, in a metapopulation context, the appropriate solution to the SLOSS debate mainly depends on the connectivity between sub-populations (i.e. flocks of the different breeders), which should be strong for multiple sub-populations to be viable [46]. While this appeared to be the case for the MAR breed based on the low Fst values among breeders, it was not the case for the HER and MAN flocks which were isolated from each other, indicated by large Fst between flocks. In addition, the HER and MAN individual flocks were of very limited size, which made them prone to genetic drift and inbreeding. This was confirmed by their longer ROH that indicate recent inbreeding, which was not the case for the MAR breed. Thus, in the case of local chicken populations, central management of a large flock is recommended from a conservation perspective, unless the flocks are sufficiently connected through the exchange of breeding animals. This was the case for the Marans breed and the CON breed, which each consist of several flocks that seemed to be sufficiently connected to maintain sufficient gene flow to limit both inbreeding and genetic drift. However, all the Marans individuals still clustered together, which confirms that the breed concept remains meaningful even when different varieties are managed by a network of fancy breeders. This is of particular importance for future conservation plans of genetic resources, since the scale at which genetic diversity should be monitored and the base unit to be conserved are crucial for decision-making.

Combining the information available for Group 1 allowed us to evaluate the management programs and the relevant parameters to consider in the future. In particular, we found a significant positive correlation between the number of generations since the start of the program and the current effective population size based on pedigree data, even when correcting for the initial effective population size at the start of the program. This correlation revealed effective management to limit inbreeding across generations, with smaller ROH and thus older inbreeding in populations with an earlier start of the management program. Obviously, the success of such a population management program relies also largely on the initial diversity in the population, as revealed by the positive correlation between initial and current effective population sizes or length of ROH. We also found that the number of sires was the most limiting factor, since it determines the number of families in the mating plan, while there were in general more females than males at the start of the program in each breed. Thus, in a hierarchical mating plan (i.e. one sire for several hens), as those used in poultry breeding, the number of males is the most limiting factor with the strongest impact on the level of inbreeding, since they are responsible for the genetic bottleneck of the population. This was confirmed by the current sex-ratio being more biased toward females than the initial sex-ratio. Thus, the focus should be on conservation of the maximum number of families, in particular in terms of sires.

### On the interest of ROH for conservation genetics

While Fis informs us about the management of populations given the allelic diversity of the population, Fit allows us to measure the absolute genetic diversity of population as a deviation from its expectation based on approximate ancestral allele frequencies. Indeed, we found a positive correlation of Fit (and a weaker one of Fis) with the proportion of fixed alleles, similarly to what Muir et al. [11] observed between the proportion of missing alleles and the inbreeding coefficient. This means that this Fit-like estimator is a good index to evaluate genetic diversity conserved in populations at the species level, which results from the effects of size of the selection nucleus, founder effect, and mating plans. In the current study, the mean Fit value was 0.26 and ranged from 0.11 to 0.46. These values are very similar to average inbreeding coefficients estimated from ROH, F-ROH, which ranged from 0.13 to 0.42, with a very strong correlation between the mean Fit and F-ROH. An advantage of the ROH inbreeding estimate used in this study is that it does not rely on any assumption about allele frequencies, and thus is insensitive to the number of individuals or populations considered, in contrast to Fit, which needs estimates of allele frequencies in multiple populations. Consequently, ROH inbreeding estimates appeared to be the best indices to monitor the genetic diversity of populations, in particular without additional information, such as pedigrees or accurate estimates of allele frequencies, as is often the case with small local populations. In addition, the length of ROH was also informative about the effectiveness of the population management program. Indeed, for the local breeds that had implemented such a program (i.e. Group 1), we observed a significant and negative correlation between the mean length of ROH and the number of generations elapsed since the start of the program (Fig. 1), i.e. less recent inbreeding for breeds that benefited from a management program early on and, thus, had effective mating plans. Of course, the length of the ROH was also influenced by the number of founders at the start of the program. The trend observed with ROH length was also observed for Ne estimates based on pedigree data, confirming their potential for monitoring the genetic diversity of these populations, as shown in previous studies [47]. In addition, Caballero et al. [48] showed that ROH are very accurate for estimating inbreeding depression in populations with a limited effective population size, which is often the case with local breeds. Nevertheless, Fis estimates remain useful since they inform us about the recent impact of mating plans and inbreeding management on genetic diversity.

Given that pedigree recording can be a daily constraint, we also show that molecular indicators can be used to efficiently monitor the conservation program at planned intervals by checking inbreeding and co-ancestry. For populations that are sub-divided in multiple flocks, for instance, Lopez-Cortegano et al. [49] suggested to pay particular attention to haplotypic diversity. In addition, genotype data also allow for routine parentage assignment for populations without mating control (e.g. free range) to drive selection of reproducers for the next generation. The spectacular growth of molecular tools makes it more and more easy to genotype a large range of animal species, either domestic or wild. Thus, it becomes important to develop a standard and easy-to-use genotyping panel that is made available to all managers of small populations at a reasonable cost. Using the same genotyping platform will make it possible to compare genetic diversity and monitor conservation programs across populations and countries for coordinated management of genetic resources at a larger scale than a country or region. This is the aim of two affordable multi-species SNP chips that are currently being developed within the H2020 IMAGE project (imageh2020.eu) for cattle, sheep, goat, horse, pig, chicken, buffalo, rabbit, quail, pigeon, duck, and bee, with 10K markers for each species [50]. However, such a moderate SNP density could make it difficult to interpret ROH-based estimates, thus additional molecular analysis with more SNPs could also be necessary for some individuals.

Our results confirm that the size of the founder population is a critical parameter to conserve genetic diversity. However, a well-planned management makes it possible to maintain genetic diversity even in populations that started with a small number of founders. Indeed, Gicquel et al. [51] showed that breeds for which conservation programs were implemented with the aim to increase population size did not always demonstrate an increase in genetic variability. Thus, explicit consideration of genetic variability and identification of its determinants could help to make conservation programs more effective in the future.

## Conclusions

We have demonstrated that a high level of genetic diversity exists in a large set of French local chicken breeds and, thus, that these breeds represent potential valuable genetic resources for the future. Indeed, such a large genetic diversity is an obligatory feature to cope with global changes and achieve more sustainable production through better adaptation to variable environmental conditions. We have also shown that an appropriate population management or breeding program can reconcile moderate production performance with conservation of genetic diversity, both within and between breeds, although performance and genetic diversity of populations are generally in conflict with each other. Indeed, avoiding genetic relatedness in the mating plan limited the increase in inbreeding that is frequently observed in other fancy breeds, while maintaining numerous breeds across the country. This is due to the particular French niche markets that are associated with a strong local appropriation of breeds to a territory and top-quality products from chickens that are often raised under free-range conditions in populations for which selection pressure is mild. We have highlighted that the number of families, combined with an increase in population size, has a greater impact on inbreeding rate than sampling a large number of male and female founders. Implementing such population management relies on careful pedigree recording and organization of breeders into either one large breeding nucleus or several smaller ones that are well connected to each other. This ensures the conservation of a large part of the initial genetic diversity. Finally, we have shown the usefulness and accuracy of ROH-based estimates and molecular tools, in general, for the evaluation and routine monitoring of genetic diversity, in particular in the absence of complete pedigrees or when only few samples are available, which is often the case for local breeds.

## Supplementary Information

**Additional file 1: Table S1.** Within-population genetic diversity estimates; raw data for Figs. 1, 2 and 3, in the main text.

**Additional file 2: Table S2.** Marginal estimated means of each diversity index per group of populations. The letters and grey color stand for significant pairwise differences between groups (Tukey correction for multiple tests).

**Additional file 3: Figure S1.** Correlation matrix of population molecular or demographic diversity indices for breeds involved in a management program (Group 1). Values correspond to Pearson’s correlation coefficients. Colored cells stand for significant correlations (p < 0.05) either positive (blue) or negative (red), the intensity depending on the strength of the correlation between estimates. White cells represent non-significant correlations. Nb.Generation stands for the number of generations since the start of the management program and Fixed for the proportion of fixed alleles.

**Additional file 4: Figure S2.** Matrix of pairwise Fst between populations. Darker blue indicates a stronger pairwise Fst.

**Additional file 5: Figure S3.** Mean assignment probability of populations to each of the two clusters. The Asian and European clusters are colored in red and blue, respectively.

**Additional file 6: Figure S4.** Unrooted neighbor-joining tree of the Hergnie breed. The colors of the surrounding circle represent the different breeders.

## Data Availability

Genomic data are available in the *recherche.data.gouv.fr* platform with following identifier https://doi.org/10.57745/67LET9.
