# Supplementary material for: Managing genetic diversity in breeding programs of small populations: the case of French local chicken breeds
Source: Genet Sel Evol. 2022 Aug 3;54:56. doi: 10.1186/s12711-022-00746-2 (PMC9347113; doi:10.1186/s12711-022-00746-2)
Supplement: Supplementary file 2 — Additional file 2: Table S2. Marginal estimated means of each diversity index per group of populations. The letters and grey color stand for significant pairwise differences between groups (Tukey correction for multiple tests). [file 12711_2022_746_MOESM2_ESM.docx]

**Table S5.** Marginal estimated means of each diversity index per group of populations. The letter and grey color stand for significant pairwise differences with other groups (Tukey correction for multiple tests).

|  | **Groups** | | |
| --- | --- | --- | --- |
| **Indices** | **Group 1** | **Group 2** | **Group 3** |
| **Fit** | Mean = 0.278  SE = 1.95.10-2  a | Mean = 0.260  SE = 4.36.10-2  a | Mean = 0.191  SE = 4.36.10-2  a |
| **Fis** | Mean = -1.37.10-2  SE = 8.34.10-3  a | Mean = 6.30.10-2  SE = 1.86.10-2  b | Mean = -1.82.10-2  SE = 1.86.10-2  a |
| **Ho** | Mean = 0.340  SE = 3.14.10-3  a | Mean = 0.316  SE = 7.02.10-3  b | Mean = 0.353  SE = 7.02.10-3  a |
| **He** | Mean = 0.336  SE = 2.13.10-3  a | Mean = 0.337  SE = 4.76.10-3  a | Mean = 0.346  SE = 4.76.10-3  a |
| **MAF** | Mean = 0.201  SE = 5.96.10-3  a | Mean = 0.224  SE = 1.33.10-2  a | Mean = 0.226  SE = 1.33.10-2  a |
| **Fixed** | Mean = 0.204  SE = 1.90.10-2  a | Mean = 0.116  SE = 4.24.10-2  a | Mean = 0.139  SE = 4.24.10-2  a |
| **F-ROH** | Mean = 0.241  SE = 1.68.10-2  a | Mean = 0.233  SE = 3.76.10-2  a | Mean = 0.211  SE = 3.76.10-2  a |
| **Length ROH** | Mean = 80.6  SE = 5.06  a | Mean = 52.9  SE = 11.31  a | Mean = 78.3  SE = 11.31  a |
| **Number of ROH** | Mean = 2780  SE = 86.4  a | Mean = 3988  SE = 193.1  b | Mean = 2458  SE = 193.1  a |
