# Supplementary figures and images for: Managing genetic diversity in breeding programs of small populations: the case of French local chicken breeds
Source: Genet Sel Evol. 2022 Aug 3;54:56. doi: 10.1186/s12711-022-00746-2 (PMC9347113; doi:10.1186/s12711-022-00746-2)

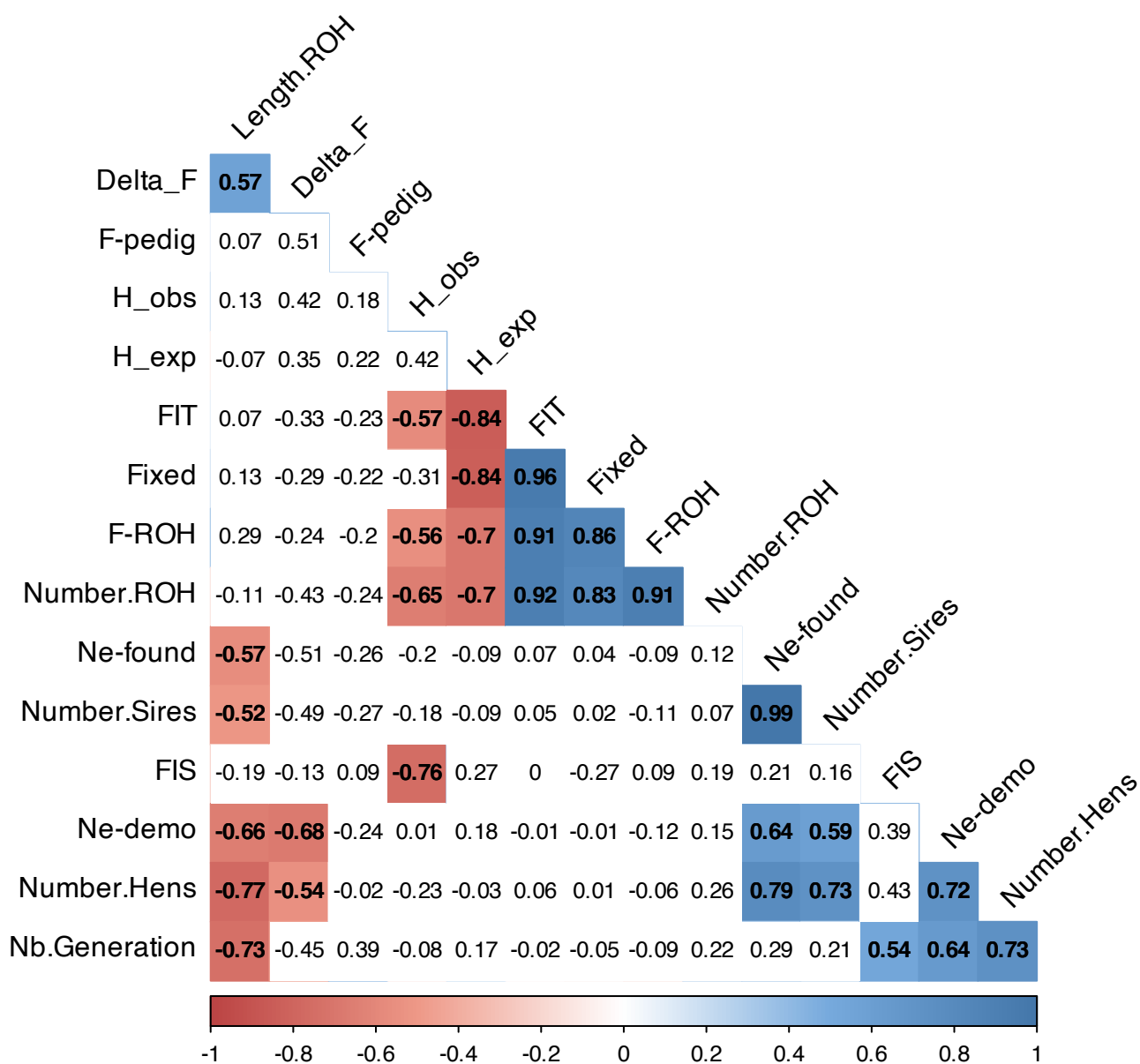

Supplement: Supplementary file 3 — Additional file 3: Figure S1. Correlation matrix of population molecular or demographic diversity indices for breeds involved in a management program (Group 1). Values correspond to Pearson’s correlation coefficients. Colored cells stand for significant correlations (p < 0.05) either positive (blue) or negative (red), the intensity depending on the strength of the correlation between estimates. White cells represent non-significant correlations. Nb.Generation stands for the number of generations since the start of the management program and Fixed for the proportion of fixed alleles. [file 12711_2022_746_MOESM3_ESM.pdf]

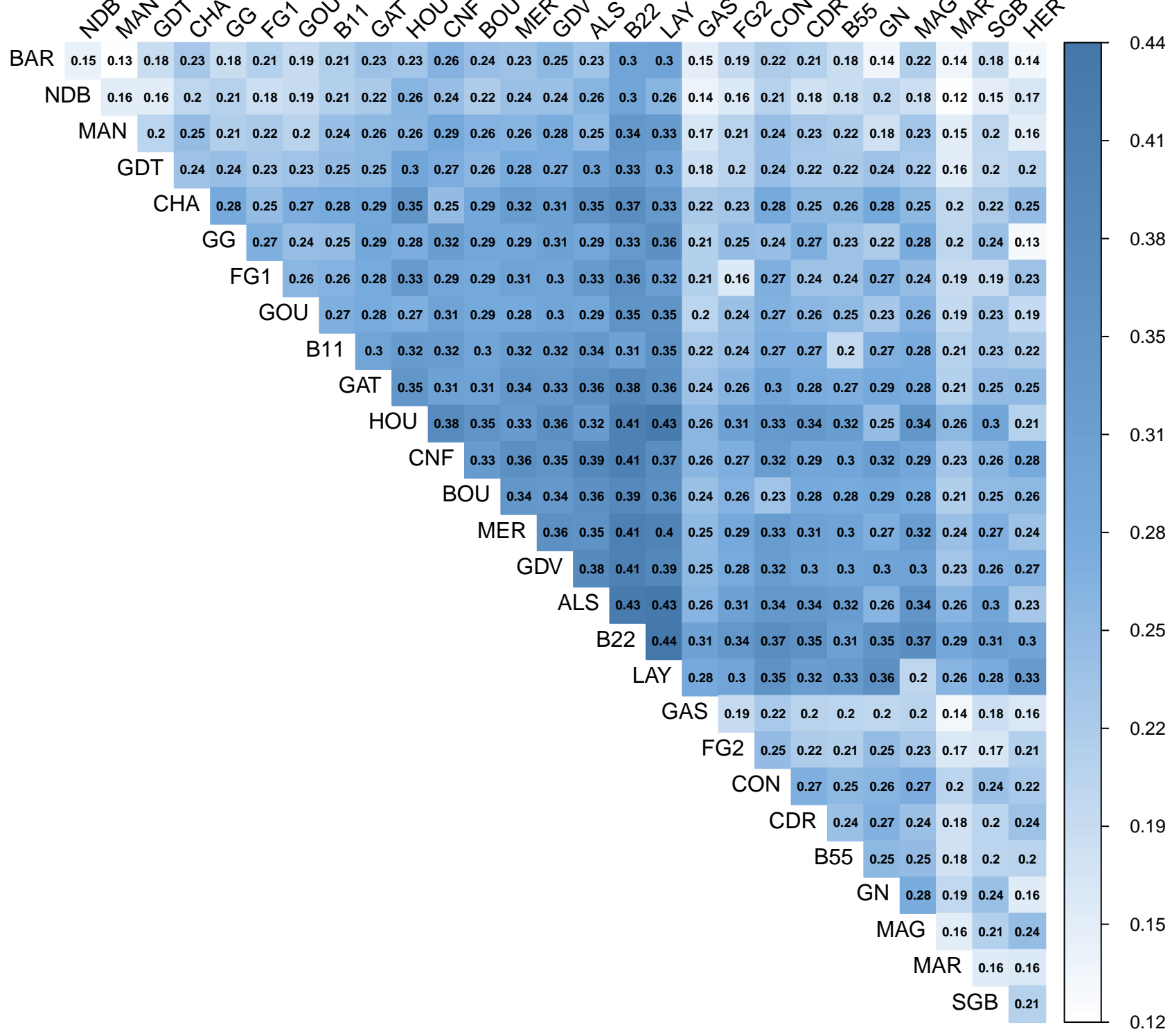

Supplement: Supplementary file 4 — Additional file 4: Figure S2. Matrix of pairwise Fst between populations. Darker blue indicates a stronger pairwise Fst. [file 12711_2022_746_MOESM4_ESM.pdf]

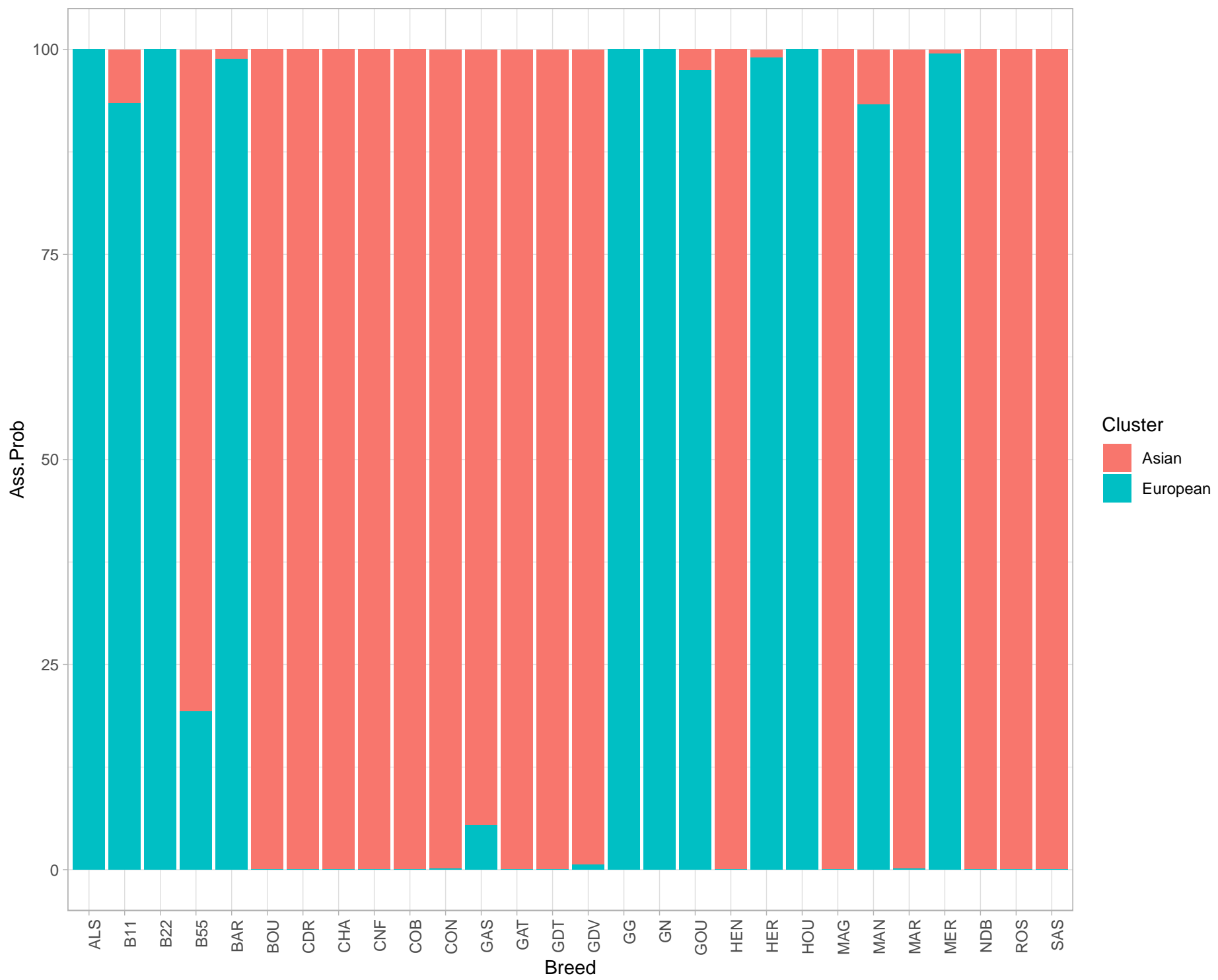

Supplement: Supplementary file 5 — Additional file 5: Figure S3. Mean assignment probability of populations to each of the two clusters. The Asian and European clusters are colored in red and blue, respectively. [file 12711_2022_746_MOESM5_ESM.pdf]

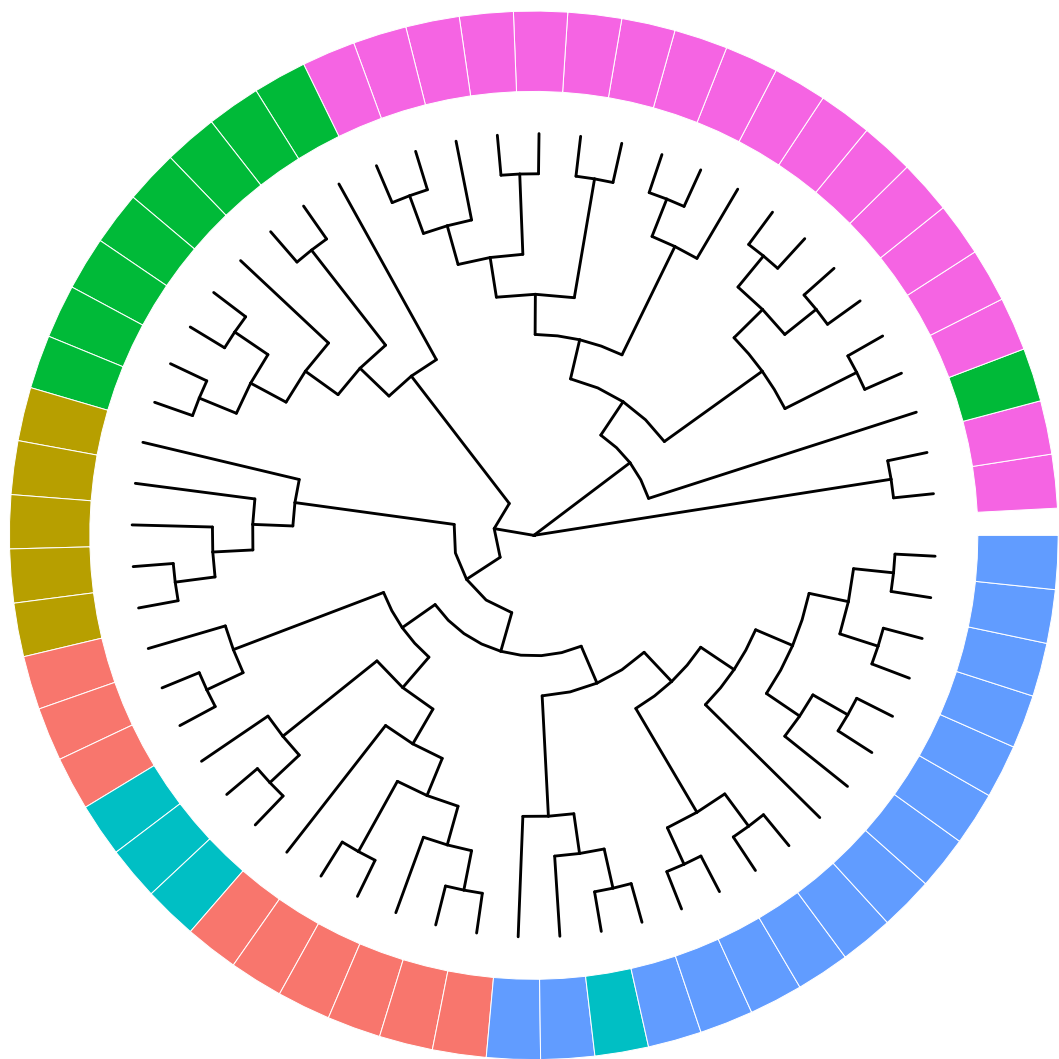

Breeder

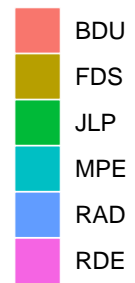

Supplement: Supplementary file 6 — Additional file 6: Figure S4. Unrooted neighbor-joining tree of the Hergnie breed. The colors of the surrounding circle represent the different breeders. [file 12711_2022_746_MOESM6_ESM.pdf]
